# HTSinfer: inferring metadata from bulk Illumina RNA-Seq libraries

**DOI:** 10.1093/bioinformatics/btaf076

**Published:** 2025-02-19

**Authors:** Máté Balajti, Rohan Kandhari, Boris Jurič, Mihaela Zavolan, Alexander Kanitz

**Affiliations:** Biozentrum, University of Basel, Basel 4056, Switzerland; Swiss Institute of Bioinformatics, Lausanne 1015, Switzerland; Biozentrum, University of Basel, Basel 4056, Switzerland; Institute of Computer Science, Masaryk University, Brno 60200, Czech Republic; Biozentrum, University of Basel, Basel 4056, Switzerland; Swiss Institute of Bioinformatics, Lausanne 1015, Switzerland; Biozentrum, University of Basel, Basel 4056, Switzerland; Swiss Institute of Bioinformatics, Lausanne 1015, Switzerland

## Abstract

**Summary:**

The Sequencing Read Archive is one of the largest and fastest-growing repositories of sequencing data, containing tens of petabytes of sequenced reads. Its data is used by a wide scientific community, often beyond the primary study that generated them. Such analyses rely on accurate metadata concerning the type of experiment and library, as well as the organism from which the sequenced reads were derived. These metadata are typically entered manually by contributors in an error-prone process, and are frequently incomplete. In addition, easy-to-use computational tools that verify the consistency and completeness of metadata describing the libraries to facilitate data reuse, are largely unavailable. Here, we introduce HTSinfer, a Python-based tool to infer metadata directly and solely from bulk RNA-sequencing data generated on Illumina platforms. HTSinfer leverages genome sequence information and diagnostic genes to rapidly and accurately infer the library source and library type, as well as the relative read orientation, 3′ adapter sequence and read length statistics. HTSinfer is written in a modular manner, published under a permissible free and open-source license and encourages contributions by the community, enabling easy addition of new functionalities, e.g. for the inference of additional metrics, or the support of different experiment types or sequencing platforms.

**Availability and implementation:**

HTSinfer is released under the Apache License 2.0. Latest code is available via GitHub at https://github.com/zavolanlab/htsinfer, while releases are published on Bioconda. A snapshot of the HTSinfer version described in this article was deposited at Zenodo at 10.5281/zenodo.13985958.

## 1 Introduction

The NCBI’s Sequencing Read Archive (SRA) (Sayers *et al.* 2022) is among the largest publicly available repositories for high-throughput sequencing data. As of November 2024, SRA contains millions of bulk RNA-Seq libraries, with 3 160 912 entries corresponding to the search criteria ((((“illumina”[Platform]) AND “rna seq”[Strategy]) AND “transcriptomic”[Source])) NOT (“scRNA-seq” OR “single-cell”). The metadata describing such samples are still mostly entered manually during sample submission, a cumbersome and error-prone process, which can lead to incorrect sample annotations. Additionally, metadata crucial for downstream analyses such as differential transcript/gene expression or gene set enrichment are not reported, because corresponding fields in the submission forms are either absent, or their provision is optional. Missing, incomplete, or inaccurate metadata can compromise analyses and lead to wrong conclusions. A small number of tools are available for inferring specific parts of sample metadata, such as the Sequence Taxonomic Analysis Tool (STAT) (Katz *et al.* 2021) for the source of the sequencing library, GUESSmyLT (Wik *et al.* 2019) for the library type, or Salmon (Patro *et al.* 2017) for the library orientation. A summary table of these tools and their specific functions was deposited at Zenodo (European Organization For Nuclear Research and OpenAIRE 2013, Balajti *et al.* 2024a). However, a comprehensive and easy-to-use solution for the inference of key metadata necessary for commonly used downstream analyses is still missing.

Here, we present HTSinfer, a Python-based application for the rapid inference of key metadata necessary for downstream analyses directly from the read libraries. Specifically, the tool enables the identification of the library’s source organism, the type of the library (single/paired-end, strandedness, and relative orientation of the reads), length statistics for the sequenced reads, and the 3′ adapter sequence. While in its default configuration HTSinfer operates with minimal input from the user, the tool is also highly configurable, allowing expert users to adjust and expand it to better suit their use cases, e.g. in workflows that automate and verify the metadata inference prior to data analysis (Katsantoni *et al.* 2024).

## 2 Implementation

**Figure 1.**
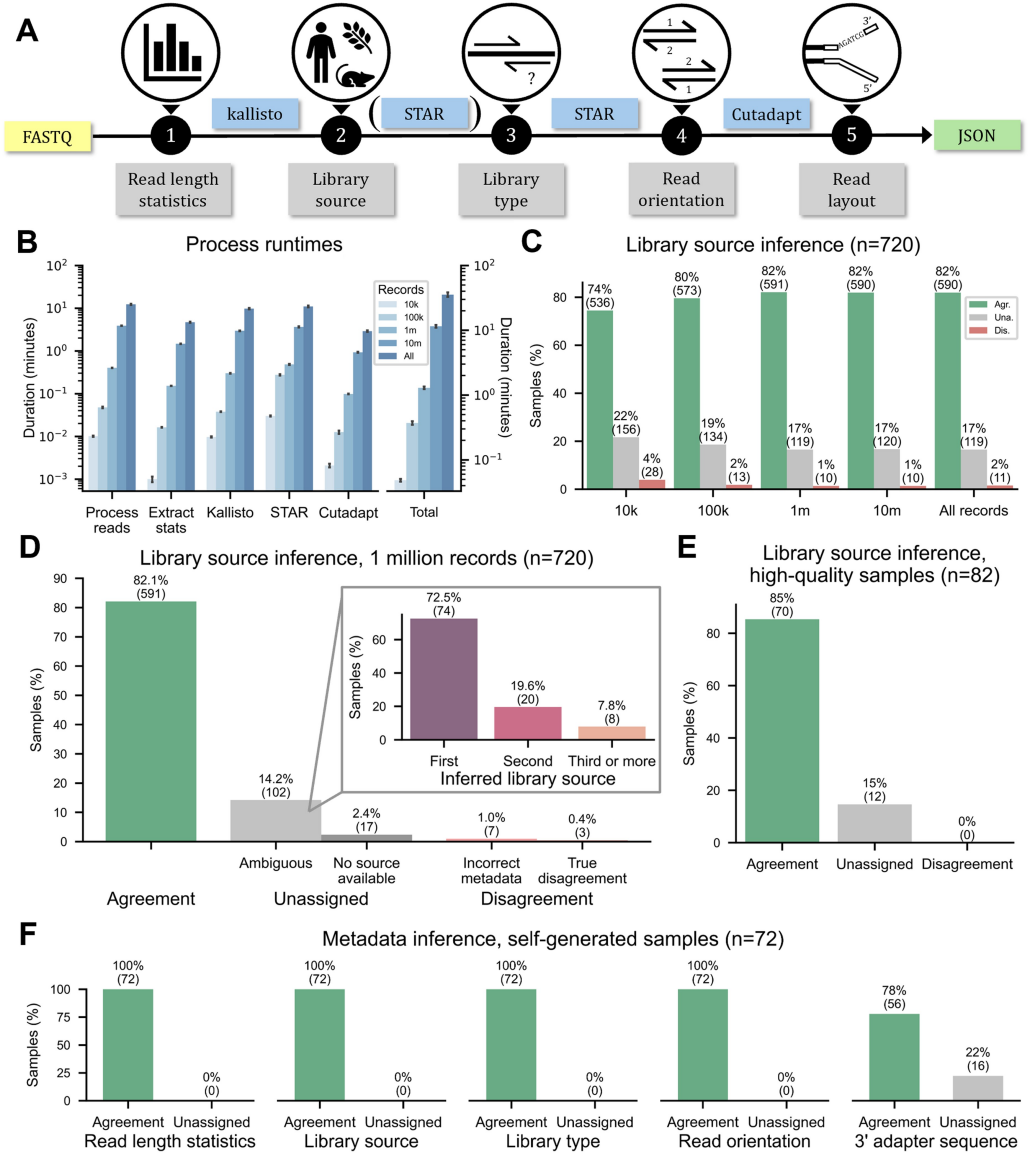
Summary of HTSinfer and case study results. In comparisons with known metadata in panels (C), (D), (E), and (F), we assigned the label “Agreement” if the HTSinfer-inferred metadata is consistent with the known metadata, and “Disagreement” otherwise. If HTSinfer did not infer metadata at all, we assigned “Unassigned” instead. Since HTSinfer determines the library source independently for each mate file in paired-end samples, in (D), (E), and (F) we assigned “Disagreement” if either of the mate files did not match the reference. (A) Overview of HTSinfer workflow, from the FASTQ file of sequencing reads to the JSON output of metadata. The tools used to infer specific metadata (indicated in the gray boxes) are shown in the boxes with blue background. (B) Runtime (in min) for analyzing SRA samples, as a function of sample size. Means and standard deviations are shown (*n* = 720 samples with 1 run each). (C) Results of library source inference for the 720 SRA samples as a function of the number of randomly selected records. (D) Detailed results from the library source inference of the SRA samples (*n* = 720), using 1 million records per sample. Samples for which there was disagreement on the library source, or the library source was considered ambiguous were further inspected. Samples for which an unambiguous call was not made were further separated into categories depending on whether the species of origin had rank 1, 2, or 3 or more based on the number of RP-mapped reads. (E) Results of library source inference on SRA samples with minimal RNA degradation (median TIN score > 70, *n* = 82). (F) Validation of results on the 72 samples generated in-house. As there were no disagreements with known metadata, the “Disagreement” columns were omitted.

HTSinfer is implemented as a Python library with a command-line executable. A high-level overview of HTSinfer’s modular design is provided in Fig. 1A. Inputs are gzipped or unzipped FASTQ files of sequenced RNA-Seq libraries, one in the case of single-end samples, or two in the case of (suspected) paired-end samples. The inferred metadata is written to STDOUT in JSON format for easy post-processing. Logging information is written to STDERR. The metadata inferred and reported by HTSinfer is described below:

### 2.1 Read length statistics

Read length statistics (minimum, maximum, mean, median, and mode) of the input FASTQ files are tabulated. These metrics provide essential parameters for downstream tools such as STAR (Dobin *et al.* 2013) and Salmon (Patro *et al.* 2017), which rely on read length information to accurately index the genome/transcriptome. By comparing minimum and maximum read lengths one can also verify that all reads are of equal length, which is necessary for certain tools, e.g. MISO (Katz *et al.* 2010), which analyzes splice isoforms. Finally, assessing the read length statistics can also reveal whether the data has already undergone some degree of processing, as libraries generated by Illumina machines generally produce reads of uniform length.

### 2.2 Library source

To reliably identify the source organism of the library, HTSinfer leverages the availability of ribosomal protein (RP) gene annotations available in public repositories. RP-encoding transcripts are highly abundant in the transcriptome of any species and should be reliably captured in RNA-Seq data (Thorrez *et al.* 2008, Petibon *et al.* 2021). For the inference of the species of origin we selected RP genes of the small ribosomal subunit that are highly conserved across Bacteria, Archaea, and Eukarya (Ban *et al.* 2014, Scarpin *et al.* 2023). To construct a reference database, the homologous genes of the human genes across species in the Ensembl databases (Harrison *et al.* 2024) were identified. Organisms with at least five RP homologous genes were selected, and the coding sequences of all the transcript isoforms were downloaded. Currently, HTSinfer uses a set of RP-encoding mRNA sequences from 385 organisms. The abundances of these transcripts are estimated with kallisto (Bray *et al.* 2016), yielding, for each input file, a table containing entries for each tested species, namely short name and taxon identifier (e.g. “hsapiens,” 9606), and total estimated expression (in transcript abundance counts) of all RP transcripts of the species. The table is then sorted by the total expression in descending order, and the species with the highest total RPM, which thus provides the strongest evidence of RP expression, is considered to be the source for the sample. Configurable cutoffs for minimal RP expression levels and for the ratio between the best and second-best-matching species are applied to minimize false assignments (e.g. when the true source organism is not covered by HTSinfer’s RP library). As subsequent inferences of library type, read orientation, and layout all depend on the correct identification of the sample source, and given the fact that the organism of the sample is known or annotated more often than not, a command-line parameter for setting the library source manually using the taxon identifier of one of the 385 currently supported organisms, is available (e.g. --tax-id = 9606).

### 2.3 Library type

Next, HTSinfer determines the type of library, i.e. whether the input files were sequenced as single- or paired-end. If two input files are provided, HTSinfer attempts to verify whether they contain mate pairs from the same paired-end library. Conversely, if only one input file is provided, HTSinfer checks whether it represents a complete single-end library, or if it contains just one set of mates from a paired-end library. It does so by first checking the sequence identifiers of the reads. The tool is optimized for the systematic identifiers used by Illumina sequencing devices, ensuring compatibility with all libraries generated by these. If mate information is not encoded in the sequence identifiers in a supported format or is absent altogether, the reads are aligned to ribosomal protein mRNAs from the appropriate organism with STAR in single-end mode. The alignments of the read pairs are then compared, to decide if they originate from a single fragment (paired-end sample) or not (single-end sample).

### 2.4 Read orientation

The library strandedness and orientation of the reads (sense or antisense relative to the direction of transcription) is then inferred from alignments to the ribosomal protein mRNAs of the source organism. If the library type was determined solely from the sequencing identifiers in the previous step, STAR aligns the reads to the ribosomal protein mRNAs of the source organism. Otherwise, the already available alignments are reused. Either way, for each alignment, the Sequence Alignment Map (SAM; Li *et al.* 2009) file generated by STAR includes specific information on whether a read sequence needed to be considered as is or as its reverse complement to compute the alignment against the corresponding transcript. In HTSinfer, we use this information to determine the library orientation (forward and reverse) essentially by counting how many reads required constructing their reverse complement, calculating their ratio among all reads and comparing the resulting value against predefined ranges of ratios for the possible outcomes. Moreover, to avoid spurious results we also require a minimum number of mapped reads supporting the determined orientation. The output notation is based on the Fragment Library Types documentation (https://salmon.readthedocs.io/en/latest/library_type.html) from Salmon. In single-end libraries, either stranded-forward (SF), stranded-reverse (SR), or unstranded (U) is assigned. In the case of paired-end libraries, HTSinfer is currently equipped to handle the “inward stranded” orientation type used by all RNA-Seq libraries sequenced on Illumina devices. Consequently, the relative orientation of mate pairs, depending on which strand the first mate originates from, is assigned either inward-stranded-forward (ISF), inward-stranded-reverse (ISR), or inward-unstranded (IU).

### 2.5 Read layout

Preparation of libraries for sequencing involves the attachment of adapter sequences. HTSinfer includes a library of eighteen 12-nucleotide-long fragments of adapter sequences, manually curated from the most common Illumina sequencing kits for RNA-sequencing (https://support-docs.illumina.com/SHARE/AdapterSequences/Content/SHARE/FrontPages/AdapterSeq.htm). The process involves iterating through the list of 3′ adapter sequences and searching for each sequence within the library sequence. This is achieved using the Aho-Corasick algorithm, which allows for efficient exact matching of multiple patterns within the reads. The most commonly identified adapter along with its corresponding frequency in the library are included in HTSinfer’s report. Similar to the procedure we apply for the inference of the library source, here we also apply configurable cutoffs for minimal frequency, as well as for the ratio between the best and second-best matches for adapter sequences. Lastly, Cutadapt (Martin 2011) is used to detect and quantify the presence of poly(A) tails in the library. The fraction of reads containing poly(A) tails is then parsed from the Cutadapt report, and is reported along the 3′ adapter sequence in the final output.

## 3 Case study

To test HTSinfer’s usability and accuracy, we downloaded from the Sequence Read Archive (SRA) (Sayers *et al.* 2022) a diverse set of 720 single- and paired-end libraries prepared from 65 distinct source organisms. Acknowledging that the processing of transcriptomic data can be computationally expensive and time-consuming, HTSinfer supports the command-line option --records to limit the number of records to consider. Mutating this parameter across the set of 720 SRA samples, we observed roughly linear scaling of runtimes with the number of records considered (Fig. 1B). As SRA does not systematically collect information on read orientation or adapter sequences, we initially focused on evaluating the agreement between the inferred and reported library sources. Importantly, we found that 1 million records are sufficient to maximize HTSinfer’s library source inference accuracy (Fig. 1C); hence, we set one million as the default value for the --records parameter.

Subjecting the library source inference for one million records to further analysis (Fig. 1D), we found agreement with the known metadata for 591 (82.1%) and disagreement for 10 (1.4%) samples. Upon further inspecting the disagreements, we found that for seven samples the library source recorded on SRA is likely misannotated or misleading (e.g. metagenomic analysis, xenografts, contamination). In 102 of the 119 samples (16.6%) for which HTSinfer did not infer a library source at all, the correct library source was among the three organisms with the highest total RP transcript abundance, but the ratio between the top and next best candidate was too small (<2) to confidently assign the library source (Fig. 1D, inset). For the remaining 17 samples, RP transcripts were not detected for any organism in our database, likely due to the experimental design (e.g. antibody repertoire sequencing) selecting against such transcripts. We found the degree of RNA integrity of a sample, as measured by the transcript integrity number (TIN) (Wang *et al.* 2016), not to be a factor considerably affecting the library source inference accuracy, as we obtained similar results whether we analyzed the entire set of 720 samples or only the 82 for which the TIN was >70 (Fig. 1E).

Read length statistics and library types inferred by HTSinfer are largely in agreement with the reported metadata, with a few exceptions (e.g. samples from a study that shows evidence of processing prior to SRA submission, follow-up issues resulting from missing library source). See supplementary table for details (Balajti *et al.* 2024a).

To systematically evaluate its performance on all of its functionalities, we tested HTSinfer on a set of 72 in-house-generated RNA-Seq libraries, for which the complete set of metadata that the tool is equipped to infer was known beforehand. Here, the library source and layout, read orientation, and read length statistics were always inferred correctly by HTSinfer (Fig. 1F). The 3′ adapter sequence was correctly inferred for 78% of the samples, and unassigned for the remaining samples, either because the frequency of adapter inclusion was very low, or the library contained evidence of one or more other common adapters in comparable frequencies. No incorrect assignments were made.

Sample and result tables for both use cases were deposited at Zenodo (European Organization For Nuclear Research and OpenAIRE 2013, Balajti *et al.* 2024a).

## 4 Discussion

HTSinfer provides a way to infer metadata from bulk RNA-Seq data, simplifying the automation of data analysis. The HTSinfer code repository, together with a quick start guide, is available on GitHub at https://github.com/zavolanlab/htsinfer. A snapshot of the specific version described in this application note is deposited at Zenodo (European Organization For Nuclear Research and OpenAIRE 2013, Balajti *et al.* 2024b). HTSinfer is also available via the Conda package manager, as part of the Bioconda channel (Grüning *et al.* 2018) at https://anaconda.org/bioconda/htsinfer. Docker images for the tool are automatically built by Bioconda and are available from BioContainers (Bai *et al.* 2021) at https://quay.io/repository/biocontainers/htsinfer. Full documentation, including installation, and usage instructions, examples and API documentation, are available at https://htsinfer.readthedocs.io.

HTSinfer adheres to good open source software development practices (Jiménez *et al.* 2017) by utilizing a free and open-source license approved by the Open Source Initiative (OSI), and by promoting transparency and collaboration. The development of HTSinfer has been open to the public from day one, inviting community contributions and encouraging users to participate in improving and expanding the tool. Unit tests are executed regularly via continuous integration (CI) to ensure stability over release versions. Additionally, continuous deployment (CD) practices are implemented to streamline the release process.

HTSinfer is an integral part of the command line interface for the ZARP RNA-Seq data analysis workflow (Katsantoni *et al.* 2024). Missing metadata for the input samples of the workflow are inferred by HTSinfer, thus allowing ZARP users to process RNA-Seq libraries even when sample metadata is not available.

While HTSinfer offers robust functionality, there are several limitations to consider. First, the tool is currently compatible only with Linux and macOS operating systems. Second, HTSinfer expects bulk RNA-Seq input files generated from Illumina sequencing platforms, which may restrict its applicability to datasets generated from other sequencing platforms or methods. Third, the inference of parameters library type, read orientation, and read layout is dependent upon the accurate inference of the library source, which can lead to inaccuracies if the library source is inferred incorrectly, or not provided beforehand.

To increase the accuracy of the library source inference, future enhancements could focus on expanding taxonomic classification to finer levels, e.g. family- and genus-level. Addressing currently missing features, such as the inference of metrics describing the fragment length distribution, the implementation of probabilistic models for estimating inference accuracy, and the expansion of the tool to different types of sequencing libraries is also a priority for future development.

## Data Availability

Supplementary materials to this article, including the sample tables for testing HTSinfer (with identifiers to the corresponding data in the Sequence Read Archive), the test results underlying the data in Figure 1, and a table comparing HTSinfer with other published tools for inferring RNA-seq sample metadata, were published on Zenodo, available at 10.5281/zenodo.14177729.

## References

[btaf076-B1] Bai J , Bandla C, Guo J et al BioContainers registry: Searching bioinformatics and proteomics tools, packages, and containers. J Proteome Res 2021;20:2056–61. 33625229 10.1021/acs.jproteome.0c00904 PMC7611561

[btaf076-B2] Balajti M , Kandari R, Jurič B et al HTSinfer: Supplementary materials. *Zenodo.* 2024a. 10.5281/zenodo.14177729

[btaf076-B3] Balajti M , Kanitz A, Kandhari R et al zavolanlab/htsinfer: v1.0.0-rc.1. *Zenodo*. 2024b. 10.5281/zenodo.13985958

[btaf076-B4] Ban N , Beckmann R, Cate JHD et al A new system for naming ribosomal proteins. Curr Opin Struct Biol 2014;24:165–9. 24524803 10.1016/j.sbi.2014.01.002 PMC4358319

[btaf076-B5] Bray NL , Pimentel H, Melsted P et al Near-optimal probabilistic RNA-seq quantification. Nat Biotechnol 2016;34:525–7. 27043002 10.1038/nbt.3519

[btaf076-B6] Dobin A , Davis CA, Schlesinger F et al STAR: ultrafast universal RNA-seq aligner. Bioinformatics 2013;29:15–21. 23104886 10.1093/bioinformatics/bts635 PMC3530905

[btaf076-B7] European Organization For Nuclear Research. OpenAIRE. *Zenodo.* 2013. https://doi.org/10.25495/7GXK-RD71

[btaf076-B8] Grüning B , Dale R, Sjödin A et al Bioconda: sustainable and comprehensive software distribution for the life sciences. Nat Methods 2018;15:475–6. 29967506 10.1038/s41592-018-0046-7 PMC11070151

[btaf076-B9] Harrison PW , Amode MR, Austine-Orimoloye O et al Ensembl 2024. Nucleic Acids Res 2024;52:D891–9. 37953337 10.1093/nar/gkad1049 PMC10767893

[btaf076-B10] Jiménez RC , Kuzak M, Alhamdoosh M et al Four simple recommendations to encourage best practices in research software. F1000Res 2017;6:876. 10.12688/f1000research.11407.1 PMC5490478 28751965

[btaf076-B11] Katsantoni M , Gypas F, Herrmann CJ et al ZARP: A user-friendly and versatile RNA-seq analysis workflow. F1000Res 2024;13:533.

[btaf076-B12] Katz KS , Shutov O, Lapoint R et al STAT: A fast, scalable, minhash-based *k*-mer tool to assess Sequence Read Archive next-generation sequence submissions. Genome Biol 2021;22:270. 34544477 10.1186/s13059-021-02490-0 PMC8450716

[btaf076-B13] Katz Y , Wang ET, Airoldi EM et al Analysis and design of RNA sequencing experiments for identifying isoform regulation. Nat Methods 2010;7:1009–15. 21057496 10.1038/nmeth.1528 PMC3037023

[btaf076-B14] Li H , Handsaker B, Wysoker A et al The sequence alignment/map format and samtools. Bioinformatics 2009;25:2078–9. 19505943 10.1093/bioinformatics/btp352 PMC2723002

[btaf076-B15] Martin M. Cutadapt removes adapter sequences from high-throughput sequencing reads. EMBnet j 2011;17:10.

[btaf076-B16] Patro R , Duggal G, Love MI et al Salmon provides fast and bias-aware quantification of transcript expression. Nat Methods 2017;14:417–9. 28263959 10.1038/nmeth.4197 PMC5600148

[btaf076-B17] Petibon C , Malik Ghulam M, Catala M et al Regulation of ribosomal protein genes: An ordered anarchy. Wiley Interdiscip Rev RNA 2021;12:e1632. 33038057 10.1002/wrna.1632 PMC8047918

[btaf076-B18] Sayers EW , Bolton EE, Brister JR et al Database resources of the national center for biotechnology information. Nucleic Acids Res 2022;50:D20–6. 34850941 10.1093/nar/gkab1112 PMC8728269

[btaf076-B19] Scarpin MR , Busche M, Martinez RE et al An updated nomenclature for plant ribosomal protein genes. Plant Cell 2023;35:640–3. 36423343 10.1093/plcell/koac333 PMC9940865

[btaf076-B20] Thorrez L , Van Deun K, Tranchevent L-C et al Using ribosomal protein genes as reference: A tale of caution. PLoS One 2008;3:e1854. 18365009 10.1371/journal.pone.0001854 PMC2267211

[btaf076-B21] Wang L , Nie J, Sicotte H et al Measure transcript integrity using RNA-seq data. BMC Bioinformatics 2016;17:58. 26842848 10.1186/s12859-016-0922-z PMC4739097

[btaf076-B22] Wik E , Olin H, Haughey C et al GUESSmyLT: Software to guess the RNA-seq library type of paired and single end read files. JOSS 2019;4:1344.

